# Supplementary material for: Identification of photoperiod-induced specific miRNAs in the adrenal glands of Sunite sheep (Ovis aries)
Source: Front Vet Sci. 2022 Jul 22;9:888207. doi: 10.3389/fvets.2022.888207 (PMC9354845; doi:10.3389/fvets.2022.888207)
Supplement: Supplementary file 1 [file Data_Sheet_1.ZIP › Supplementary file/miRDeep2 file.zip!/miRDeep2 file/Know_Structure/SPLP42A3/oar-mir-376b.pdf]

miRBase precursor : oar-mir-376b
Total read count : 461
oar-miR-376e-5p read count : 173
oar-miR-376b-5p read count : 173
oar-miR-376b-3p read count : 114
remaining reads : 1

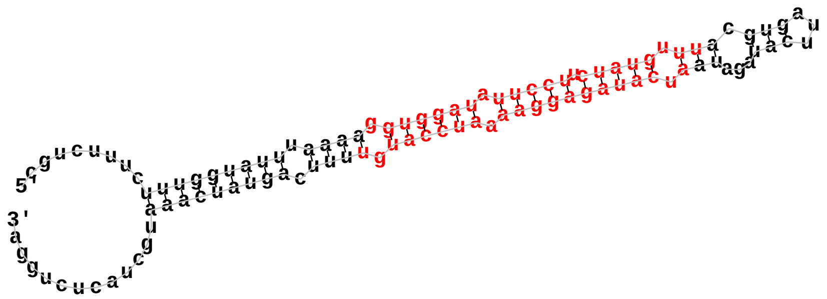

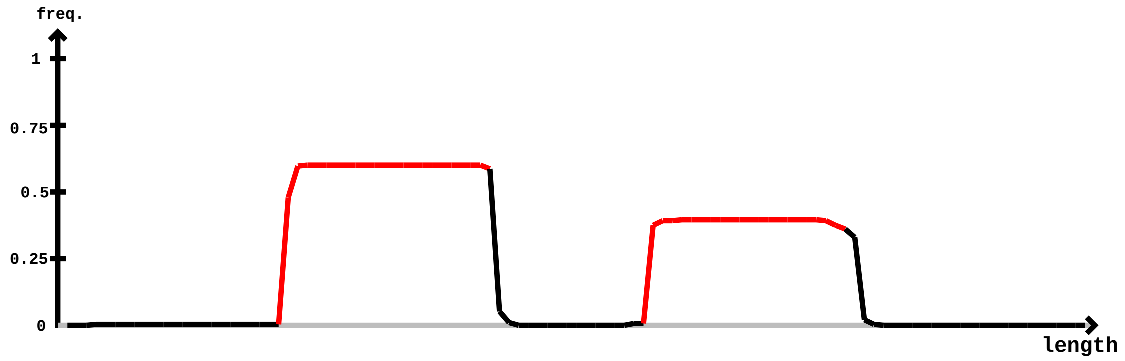


```
                                                                   oar-miR-376b-3p
                      oar-miR-376b-5p
                      oar-miR-376e-5p
5'- cgucuuucuuugguauuuaaaagguggauauuccuucuauguuuacgugauucauagauaaucauagaggaaaaauccauguuuucaguaucaaaugcuacucugga   -3'   exp
    ........((((((((.((((.((((((.(((((.(((((.(((.(((...)))...))).)))))))))).)))))).)))).))))))))...............   reads   mm   sample
    ..ucuuucuuugguauuuaaaa.....................................................................................   1       0    seq
    ......................gguggauauuccuucuauguu................................................................   4       0    seq
    ......................gguggauauuccuucuauguuu...............................................................   125     0    seq
    ......................gguggauauuccuucuauguuua..............................................................   9       0    seq
    .......................guggauauuccuucuauguuu...............................................................   28      0    seq
    .......................guggauauuccuucuauguuua..............................................................   3       0    seq
    .......................guggauauuccuucuauguuuac.............................................................   3       0    seq
    ........................uggauauuccuucuauguuu...............................................................   1       0    seq
    ..........................................................uaaucauagaggaaaaauccaug..........................   2       0    seq
    ............................................................aucauagaggaaaaaucca............................   1       0    seq
    ............................................................aucauagaggaaaaauccau...........................   5       0    seq
    ............................................................aucauagaggaaaaauccaug..........................   2       0    seq
    ............................................................aucauagaggaaaaauccaugu.........................   8       0    seq
    ............................................................aucauagaggaaaaauccauguu........................   87      0    seq
    ............................................................aucauagaggaaaaauccauguuu.......................   3       0    seq
    .............................................................ucauagaggaaaaauccaugu.........................   1       0    seq
    .............................................................ucauagaggaaaaauccauguu........................   1       0    seq
    .............................................................ucauagaggaaaaauccauguuu.......................   2       0    seq
    .............................................................ucauagaggaaaaauccauguuuu......................   1       0    seq
    ...............................................................auagaggaaaaauccauguu........................   1       0    seq
```
